# A fieldable electrostatic air sampler enabling tuberculosis detection in bioaerosols

**DOI:** 10.1016/j.tube.2019.101896

**Published:** 2020-01

**Authors:** Nuno Rufino de Sousa, Niklas Sandström, Lei Shen, Kathleen Håkansson, Rafaella Vezozzo, Klas I. Udekwu, Julio Croda, Antonio Gigliotti Rothfuchs

**Affiliations:** a Department of Microbiology, Tumor and Cell Biology (MTC), Karolinska Institutet, Stockholm, Sweden; b Faculty of Health Sciences, Federal University of Grande Dourados, Dourados, Brazil; c Department of Molecular Biosciences, Wenner-Gren Institutet, Stockholms Universitet, Stockholm, Sweden; d School of Medicine, Federal University of Mato Grosso do Sul, Campo Grande, Brazil; e Oswaldo Cruz Foundation, Mato Grosso do Sul, Campo Grande, Brazil

**Keywords:** Tuberculosis, Bioaerosols, Air sampling, Pathogen detection, Diagnostics

## Abstract

Tuberculosis (TB) infects about 25% of the world population and claims more human lives than any other infectious disease. TB is spread by inhalation of aerosols containing viable *Mycobacterium tuberculosis* expectorated or exhaled by patients with active pulmonary disease. Air-sampling technology could play an important role in TB control by enabling the detection of airborne *M. tuberculosis*, but tools that are easy to use and scalable in TB hotspots are lacking. We developed an electrostatic air sampler termed the TB Hotspot DetectOR (THOR) and investigated its performance in laboratory aerosol experiments and in a prison hotspot of TB transmission. We show that THOR collects aerosols carrying microspheres, *Bacillus globigii* spores and *M. bovis* BCG, concentrating these microparticles onto a collector piece designed for subsequent detection analysis. The unit was also successfully operated in the complex setting of a prison hotspot, enabling detection of a molecular signature for *M. tuberculosis* in the cough of inmates. Future deployment of this device may lead to a measurable impact on TB case-finding by screening individuals through the aerosols they generate.

## 1 Introduction

Tuberculosis (TB) is the leading cause of death in the world due to a single infectious agent [1]. It remains a public health concern compounded by the lack of an effective vaccine and the emergence of deadly forms of drug resistant bacilli [1]. TB spreads through air, via bioaerosols carrying *Mycobacterium tuberculosis* (*Mtb*), which are expectorated through coughing and related expiratory activities by individuals with active TB [[2], [3], [4]]. These particles are inhaled by healthy individuals resulting in pulmonary infection followed by a spectrum of clinical outcomes that place a heavy burden on the healthcare systems of the developing world. Transmission, the driving force behind the global burden of TB, is a complex process affected by microbiological, environmental and host factors [5,6]. Even the recent rise in drug-resistant TB is largely attributable to transmission of resistant clinical strains rather than the emergence of resistance following misuse or poor adherence to antibiotics [7]. Efforts to block transmission, which include environmental control, improved case-finding and diagnostics interventions, are expected to reduce the incidence of TB [8]. A comprehensive analysis of the drivers of TB transmission is currently missing. Crowding and poor ventilation appear to contribute to transmission, possibly by increasing the amount of respired air shared by individuals [6]. Consistent with this, TB hotspots are typically congregate settings such as prisons, homeless shelters, slums and refugee camps [9]. Mathematical modelling suggests that efforts to reduce transmission in hotspots are projected to have important effects on community-wide TB control [10].

Up until recently, the quantum, biophysical and bacteriologic nature of infectious aerosols remained poorly characterized due to the lack of adequate technology. Advances in such technology could be used to screen individuals through cough or populations of individuals through active surveillance of air. Recent studies highlight the success of collecting *Mtb* from bioaerosols derived from human expiratory maneuvers. These are highly quantitative and informative but may not be suited for mass implementation [4,[11], [12], [13], [14], [15]].

There are numerous, essential elements and limitations to developing a simple, scalable device for air sampling in TB-challenged geographies. An important technical aspect related to air sampling is the ability of the unit to efficiently and rapidly filter a large volume of air. Another aspect is the ability to generate a sample that can be easily processed and analyzed for *Mtb* in subsequent steps without the need for laborious sample preparation and concentration procedures. It is also critical to widely deploy the unit in TB hotspots, where resources are limited. Herein we report the development of a practical, low-cost, electrostatic air sampler for detecting tubercle bacilli. We show that our academic prototype collects aerosols carrying microspheres, bacterial spores and mycobacteria. The device was suitable for operation in TB hotspots and for detecting *Mtb* in cough by PCR. This work aligns with continued efforts to develop a scalable diagnostic approach for TB by screening infectious bioaerosols to identify contagious persons and transmission hotspots.

## 2 Materials and Methods

### 2.1 Bacteria and microspheres

*Mycobacterium bovis* Bacille Calmette-Guérin (BCG) strain Pasteur was expanded at 37 °C in Middlebrook 7H9 broth supplemented with ADC (both from BD Clinical Sciences), washed, aliquoted in PBS and stored at −80 °C until further use. Quantification of BCG Colony-forming units (CFUs) in stocks and aerosol samples was determined by culture on 7H11 agar supplemented with OADC (both from BD Clinical Sciences) at 37 °C. Lyophilized endospores of *Bacillus atrophaeus* var. *globigii* (*Bg*) were resuspended in sterile deionized (DI) water. Quantification of *Bg* CFUs in stocks and aerosol samples was determined by culture on LB Miller agar (Sigma-Aldrich) at 37 °C. One-micron, yellow-green (505/515) fluorescent, polystyrene FluoSpheres (Invitrogen) were stored at 4 °C until used.

### 2.2 THOR and its function

**Fig. 1:**
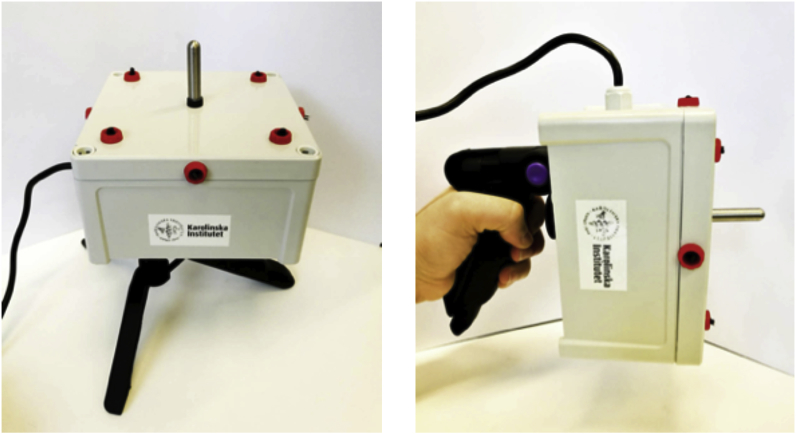
THOR electrostatic air sampler. THOR is a compact, handheld/-portable air sampler powered by standard AC 100–240 V power supply or operated from a portable battery. During THOR-*on* mode, ionization is discharged from the 4 ports (parts in red) on the top side of the unit and ionized, airborne particles are electrostatically precipitated onto the collector piece on the center, top part of the unit. The collector piece is a small replaceable, reusable, stainless steel rod.

An aerosol sampler based on corona discharge ionization and electrostatic precipitation, termed the TB Hotspot DetectOR or *THOR* was custom-built. The device (Fig. 1) has dimensions 13 cm (L) x 13 cm (W) x 7.5 cm (H), weighs 0.4 kg and consists of a drop-proof and IP66 sealed-plastic enclosure that accommodates a −20 kV high-voltage unit and a high-ohm resistor, which limits the output current. An inlet power socket is located on the side of the device and connects to a 100–240 V wall socket or external battery via an AC/DC power adapter (9 V, 360 mA). Insulated wiring connects the negative output voltage to a maximum of eight ionization ports. Replaceable carbon brush ionizers are mounted on the outside of the device in designated ports. Fewer ports may be used by closing unused ports with insulating rubber plugs. A magnetic holder on the top-center part of the device is electrically grounded via the high-voltage unit. The collector piece is a 3.5 cm long and 0.88 cm in diameter stainless-steel rod designed to fit into a 2 mL microcentrifuge tube. It is magnetically attached and electrically connected to the grounded holder on the device. A standard tripod socket on the bottom of the device enables THOR to be placed on a tripod or handle. THOR has no moving parts and operates silently at low power.

THOR is activated by connecting it to power. It generates a strong electric field between the −20 kV ionizers and the grounded collector (Supplementary Fig. 1A). A corona discharge is formed at the small radius tips of the carbon brush fibers of the ionizers and emitted electrons ionize air molecules at the vicinity of the carbon brush tips. A Townsend avalanche generates a high number of ions that also ionize aerosol particles, which then accelerate in the electric field by electrostatic forces towards the collector where they impact and lose their charge. THOR is by function both an electrostatic air pump and aerosol sampler.

In the aerosol sampling experiments described below, “THOR *on*” refers to when the device is connected to power and actively samples aerosol particles to the collector piece through ionization and electrostatic transport and precipitation, as described above. “THOR *off*” refers to when the device is not connected to power and aerosol particles passively settle onto the collector piece without the aid of electrostatic forces.

### 2.3 Aerosol sampling

Aerosol experiments were performed inside an PVC containment chamber (Solo Containment, UK) (Supplementary Fig. 2). Air was drawn into the enclosure through a HEPA-filter using an Attix 30 vacuum cleaner (Nilfisk, Sweden) to purge particles from the chamber before the start of experiments. Stock solutions of respective microparticles (BCG, *Bg* spores or FluoSpheres) were diluted in DI water to desired concentrations and aerosolized into the chamber using a 4-jet Blaustein atomizing module (BLAM) nebulizer (CH Technologies, USA). Tests with FluoSpheres established a steady concentration of particles in the enclosure after 8 min; air sampling was therefore routinely initiated 8 min after completing the aerosolization cycle on the BLAM.

THOR (Fig. 1) was located 90 cm from the ground, 100 cm away and 70 cm below the BLAM aerosol port. THOR was loaded with the collector piece, turned *on* or left *off* for 15 min. In some experiments, sterile cotton swabs (VWR) were used to sample the top, sides and wiring of the device after sampling. Where indicated, a handheld 3016 IAQ particle counter (Lighthouse, USA) was used to measure FluoSpheres.

In some experiments a Coriolis® μ cyclone sampler (Bertin Instruments, Montigny-le-Bretonneux, France) was used to collect airborne *Bg* or FluoSpheres. The Coriolis was operated at a flow rate of 300 L_air_/min. Where stated, a cumulative sampling protocol was used to circumvent issues of sample recovery during prolonged operation (our unpublished data). Briefly, a new collector cone containing 15 mL of fluid was replaced on the Coriolis every 5 min (for *Bg*) or 10 min (for FluoSpheres) for the duration of sampling. CFUs and FluoSpheres were quantified from each cone, summed and presented as the total amount of CFUs or FluoSpheres obtained.

### 2.4 Sample extraction from THOR collector piece

After air sampling, the collector piece was transferred into a microcentrifuge tube by flipping it over the collector piece and removing the two from THOR with a simple twist by the hand to break the magnetic attachment. Thereafter, 0.5 mL PBS-0.05% Tween-80 (PBS-T80) was added to the microcentrifuge tube with the collector unit inside followed by incubation at room temperature for 2 min and then vortexing for 1 min. The collector unit was then removed using a magnet and the extracted sample in liquid left in the tube.

### 2.5 Ethics statement

All participants provided written informed consent prior to study participation. The study was approved by the National Committee for Ethics in Research (Number 37237814.4.4.0000.5160). Environmental sampling was voluntary and part of a mass TB-screening effort among the incarcerated population in the Mato Grosso do Sul state, Brazil.

### 2.6 Cough sampling on THOR

Participants were asked to stand and hold THOR approximately 30 cm away from their mouth and to cough in the direction of the device for 3 min. The device was disinfected with ethanol before every sampling. Sampling was performed on 4 consecutive days. Cough collection from inmates was performed in an outdoor patio. Penitentiary agents were screened in a separate, outdoor patio or in an open room with natural ventilation off-limit to inmates. Individuals undergoing sampling were separated from other participants by a radius of at least 4 m. After sampling, THOR was handed over to research staff (wearing PPE) who secured the collector piece into a microcentrifuge tube. Material was obtained from each collector piece as described under 2.4 and subjected to DNA extraction.

### 2.7 DNA extraction

After cough sampling on THOR, DNA was extracted from the collector piece using the DNeasy Power Soil Kit (Qiagen) according to instructions of the manufacturer. A BCG standard for PCR was generated by extracting DNA from BCG grown on 7H11 agar using the DNeasy Blood and Tissue Kit (Qiagen) according to instructions of the manufacturer. The concentration of DNA in the extracted material was determined on a NanoDrop 2000c (Thermo Scientific). DNA was stored at −20 °C until further use. For laboratory aerosol experiments, sample obtained from the THOR collector piece was subjected to PCR without DNA extraction.

### 2.8 PCR for IS*6110*

Real-time TaqMan PCR was performed using a primer pair and TaqMan probe for the mycobacterial insertion sequence (IS)*6110* [16]. IS*6110* is present in variable copy number among members of the *Mtb* complex with a single copy in BCG Pasteur [17]. PCR was performed in a 7500 real-time PCR machine on MicroAmp Optical 96-well plates with TaqMan Universal Master Mix II with UNG (all from Applied Biosystems), 0.3 μM forward and reverse primers (Sigma-Aldrich) and 0.25 μM TaqMan probe (Applied Biosystems). A standard curve generated from a series dilution of a known amount of BCG DNA was included in the reaction and used to quantify the number of BCG genome equivalents in aerosol samples.

### 2.9 Scanning electron microscopy (SEM)

*Bg* or BCG sampled on THOR were extracted from the collector piece as described above and prepared for SEM. Extracted samples were fixed in 2.5% glutaraldehyde/1% paraformaldehyde in PBS and adhered onto a pore membrane. The membrane was rinsed with MilliQ water, transferred into absolute ethanol following step-wise dehydration and subjected to critical-point drying on a CPD030 dryer (Leica Microsystems). The membrane was mounted on an aluminum stub using double-sided carbon tape and sputter-coated with Platinum on a Q150T ES coating system (Quorum Technologies). The sample was then analyzed on a Gemini Ultra 55 SEM (Ziess). In some experiments the collector piece itself was fixed after air sampling and subjected to SEM.

### 2.10 Flow cytometric quantification of microspheres

FluoSpheres sampled on THOR or Coriolis were quantified on a flow cytometer using CountBright Absolute Counting Beads (Invitrogen). Briefly, a defined number of CountBright beads were added to 1 mL of sample, samples were acquired on a FACSCalibur (BD Biosciences) and analyzed on FlowJo (Tree Star). Sampling on THOR did not affect the integrity of Fluospheres, which were clearly detectable and distinguishable from counting beads (Data not shown). The concentration of FluoSpheres in the sample was calculated with CountBright beads according to the instructions of the manufacturer (Invitrogen).

### 2.11 Statistical analyses

The significance of differences in data group means was analyzed by Student's *t*-test or Anova where appropriate, with a cut-off of p < 0.05.

## 3 Results

### 3.1 A TB Hotspot Detector (THOR) that samples air through electrostatic precipitation

Our prototype TB Hotspot Detector or *THOR* is a small, portable, electrostatic air sampler that can be mounted on a tripod or handle for easy deployment in field settings (Fig. 1). The “plug-n-play” device features low-power requirements, silent operation and has a replaceable collector piece and ionizers that are easy to access. It was assembled with commercial parts costing <800 SEK (<83 USD, 2019). Finite element modelling (COMSOL Multiphysics, Sweden) illustrates the electric field generated by THOR, which is used for transport of ionized aerosol particles from air to the collector (Supplementary Fig. 1A**).** Initial testing with smoke particulates allowed for visual confirmation of active sampling onto the collector piece of the device (Supplementary Fig. 1B and Supplementary Movie 1).

Supplementary video related to this article can be found at https://doi.org/10.1016/j.tube.2019.101896.

The following is the supplementary data related to this article:

Multimedia component 1 Multimedia component 1

### 3.2 THOR collects aerosolized microparticles from air

**Fig. 2:**
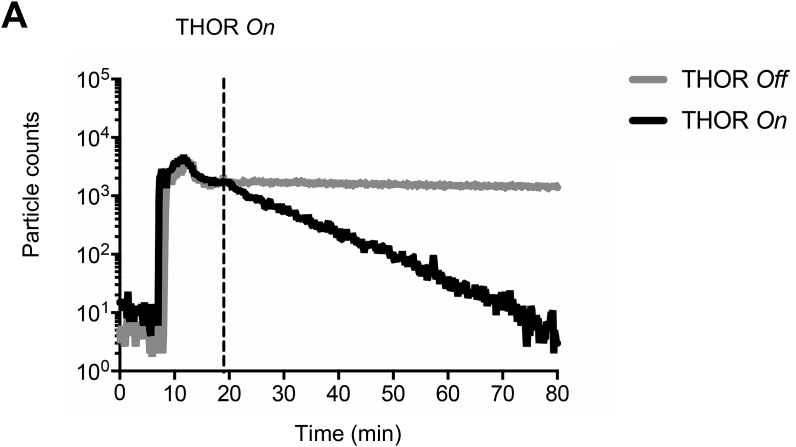
Rapid reduction of microspheres from air during active sampling on THOR. FluoSpheres (1 μm, 5 × 10^8^) were aerosolized inside the aerosol chamber (Supplementary Fig. 2). THOR (Fig. 1) was turned *on* or left *off* while a particle counter was used to record airborne microspheres in the chamber. One of 4 independent experiments shown.

To investigate more complex sampling scenarios with aerosols, a containment chamber with an inner volume of 9.3 m^3^ was tailored, assembled and placed inside a Biosafety Level-2 laboratory (Supplementary Fig. 2). As an indirect proxy for collection, we assessed the ability of THOR to “clear” microspheres from air. One-micron polystyrene spheres were aerosolized inside the chamber, THOR was activated and a particle counter used to record the number of particles in the enclosure over time. The number of microspheres detected in the air began to decay as soon as THOR was activated (Fig. 2). Particle counts decayed more than 2.5 orders of magnitude within 60 min (Fig. 2), revealing an effective clearance rate of approximately 100 L_air_/min.

**Fig. 3:**
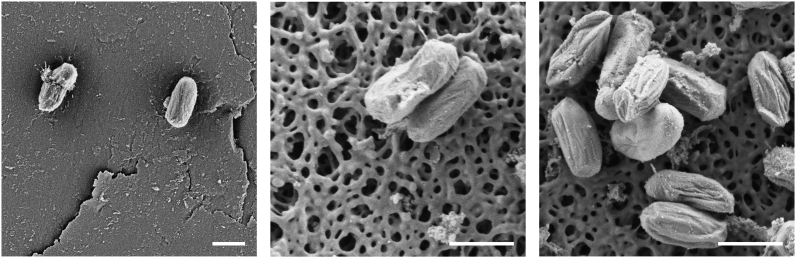
Aerosolized *Bg* sampled on THOR detected by SEM. *Bg* spores (1 × 10^8^ CFUs) were aerosolized in the chamber. Sampling on THOR was performed for 15 min and collected material subjected to SEM analysis. Electron micrographs depict *Bg* on the surface of the THOR collector piece after active sampling but before sample extraction (left panel), *Bg* extracted from the collector piece after active sampling (center panel) and *Bg* obtained from a stock preparation (right panel). Scale bars represent 1 μm. Working distance from sample was 4.5 (left panel) and 4.4 mm (center and right panels), respectively. One of 2 independent experiments shown.

Next, the ability of THOR to sample bioaerosols in the micron range was investigated using spores of the Anthrax simulant *Bacillus atrophaeus,* [18], also known as *B. globigii* (*Bg*), a benchmark agent of aerobiology work*. Bg* was consequently aerosolized in the chamber and air-sampling performed with THOR for 15 min. *Bg* was directly visualized on the collector piece by scanning electron microscopy (SEM) (Fig. 3). *Bg* was also clearly detected on material extracted from the collector piece (Fig. 3), an extraction done by just vortexing the collector in 0.5 mL buffer. These observations demonstrate that THOR can sample bioaerosols containing bacterial spores and establish a simple protocol for extracting material from the unit's collector piece.

**Fig. 4:**
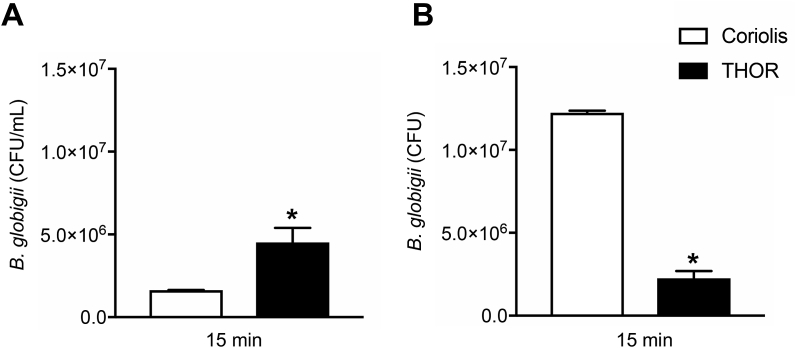
Air sampling on THOR allows collected material to be concentrated on the unit. **A** and **B**, *Bg* spores (8.4 × 10^9^ CFUs) were aerosolized and sampling performed for 15 min on THOR or Coriolis. *Bg* CFUs obtained from each sampler were quantified on LB agar and presented as a concentration (**A**) or total number of CFUs (**B**). Data from 3 experimental replicates for each group are graphed. * denotes statistically significant differences between THOR and Coriolis.

**Fig. 5:**
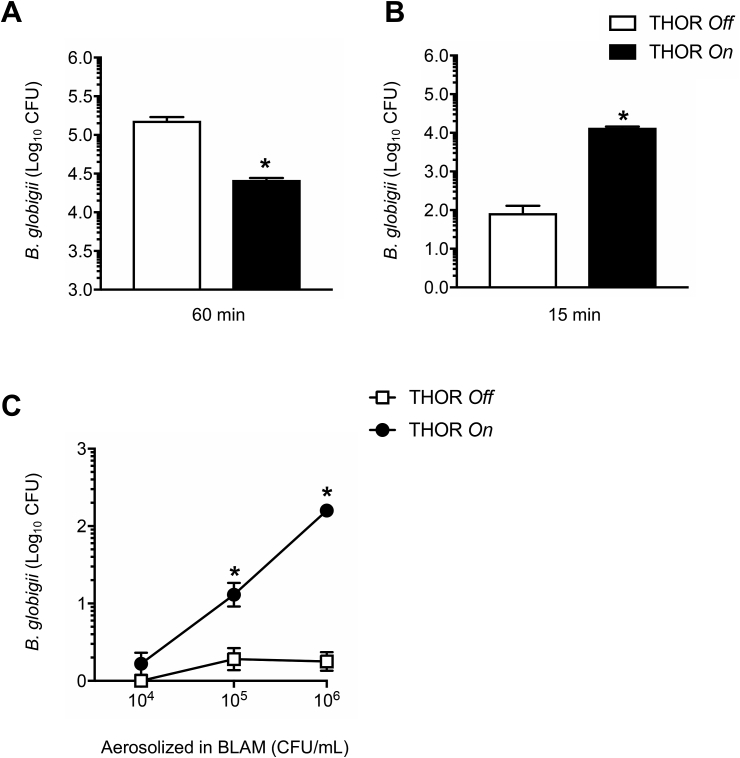
Aerosolized *Bg* sampled on THOR detected by re-growth of CFUs on agar. **A** and **B**, *Bg* spores (1.2 × 10^7^ CFUs) were aerosolized, THOR was turned *on* (THOR *On*) or left inactive (THOR *Off*) for 15 min after which *Bg* was quantified from the THOR collector piece (**B**). The Coriolis was then used to sample air from the chamber for 60 min using the cumulative sampling protocol described in Materials and Methods, to quantify remaining levels of airborne *Bg* (**A**). **C,** The given concentrations of *Bg* spores were aerosolized in the chamber, sampling on THOR was performed for 15 min and *Bg* CFUs quantified from the collector piece. Bars indicate standard error of the mean. Data from at least 3 experimental replicates for each group are graphed. * denotes statistically significant differences between THOR and Coriolis (**A**) or between THOR *On* and THOR *Off* (**B** and **C**).

We then compared THOR to the Coriolis® μ (Bertin Instruments, Montigny-le-Bretonneux, France), a state-of-the-art, cyclone-based air sampler. The Coriolis was chosen for this comparison because it is a high-volume sampler that unlike THOR collects airborne particles directly into liquid. Interestingly, aerosol experiments show that *Bg* collected with THOR and extracted into liquid was approximately 3-fold more concentrated than *Bg* collected with the Coriolis (Fig. 4A), whereas the total number of sampled *Bg* was higher in the more diluted sample from the Coriolis (Fig. 4B). Since the Coriolis can theoretically process the total air volume of the containment chamber in approximately 30 min, it was exploited in subsequent experiments to investigate THOR sampling capacity. As such, *Bg* was aerosolized in the chamber, THOR was activated for 15 min and thereafter the Coriolis was used to determine remaining *Bg* levels in the air. The number of *Bg* CFUs detected on the Coriolis were found to be reduced by almost 1 order of magnitude with THOR *on* compared with THOR *off* (Fig. 5A). Reduction in the levels of airborne *Bg* was in turn associated with increased recovery of *Bg* from the THOR collector piece (Fig. 5B). Recovery of *Bg* from the collector piece was dramatically higher with THOR *on* compared with THOR *off* (Fig. 5B), confirming active collection of bioaerosols on the unit while it is in operation. Similar results were obtained in experiments with 1-μm polystyrene spheres (Supplementary Fig. 3). Importantly, THOR was capable of actively collecting *Bg* aerosols released at a concentration of 10^5^ CFUs/mL, corresponding to detection of approximately 11 CFUs/L_air_ (Fig. 5C).

### 3.3 THOR samples aerosolized mycobacteria

**Fig. 6:**
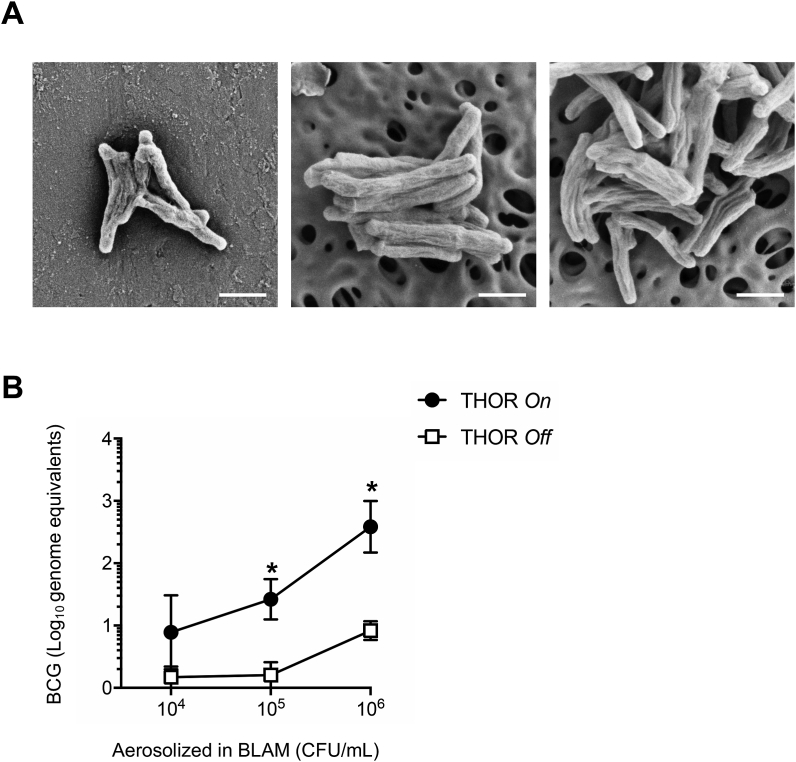
Aerosolized BCG sampled on THOR is detected by SEM and PCR. **A,** BCG (1 × 10^6^ CFUs) were aerosolized, sampling on THOR performed for 15 min and the collected material subjected to SEM analysis. Electron micrographs depict BCG on the surface of the THOR collector piece after active sampling but before extraction (left panel), BCG extracted from the collector piece after active sampling (center panel), and BCG obtained directly from 7H9 broth (right panel). Scale bars represent 1 μm. Working distance from sample was 4.5 (left panel) and 4.4 mm (center and right panels) respectively. One of 2 independent experiments shown. **B**, The given concentrations of BCG were aerosolized, sampling on THOR was performed for 15 min and collected material on THOR quantified by TaqMan PCR for IS*6110*. Material extracted from the collector piece did not generate CFUs of BCG on mycobacteria-selective 7H11 agar. Bars indicate standard error of the mean. Data from at least 3 experimental replicates for each group are graphed. One of 3 independent experiments shown. * denotes statistically significant differences between THOR *On* and THOR *Off*.

The ability of THOR to sample bioaerosols carrying mycobacteria was investigated. For this, *M. bovis* BCG was aerosolized in the chamber as a simulant for *Mtb*. Similar to results with *Bg* we found using SEM that our prototype can collect mycobacteria from air (Fig. 6A). It was not possible to re-grow mycobacteria from the collector piece, even after release of a high aerosol concentration (10^6^ CFUs/mL). On the other hand, the device was successfully used in conjunction with real-time TaqMan PCR to detect BCG from aerosols (Fig. 6B). PCR relied on the amplification of the *Mtb* complex-specific insertion sequence (IS) *6110* [16] and supported detection of BCG across a wide range of aerosolized concentrations (Fig. 6B). These results reveal PCR as a suitable method for detecting *Mtb* concurrent with THOR's electrostatic air-sampling approach. The latter is in line with previous results on the success of PCR to detect airborne *Mtb* using other air-sampling methods [[19], [20], [21], [22], [23], [24]].

### 3.4 THOR enables molecular detection of *Mtb* from cough-generated aerosols

Field-testing of the prototype was performed in a prison hotspot in Dourados, Mato Grosso do Sul state, Brazil, a congregate setting where some of the highest TB rates in contemporary time have been recorded [25]. Inmates from the Dourados State Penitentiary (PED) were asked to hold and to cough in the direction of THOR for 3 min. The penitentiary agents (guards) patrolling the facility participated as endemic controls. Following cough sampling on THOR, DNA was extracted from the collector piece and subjected to the same PCR used to detect BCG in laboratory aerosol experiments. A total of 66 inmates and 18 guards were investigated in this way (Table 1). Interestingly, all guards were negative and 12 inmates tested positive. Of the 12 inmates that tested positive with THOR, 10 of these were identified during a random screening of 61 inmates in the prison. The remaining 2 THOR-positive inmates came from a group of 5 inmates selected for investigation because of a previous history of TB. These inmates had been diagnosed with TB about 7 months before THOR sampling, had completed treatment and were smear-positive at the time of diagnosis. Two of the negative individuals were receiving TB treatment at the time of sampling but were smear-negative. The third, negative inmate was smear-positive but sampled 11 months after diagnosis. These observations demonstrate that THOR in conjunction with PCR can detect an *Mtb* IS*6110* signature from patient cough.

**Table 1:** PCR-based detection of *Mtb* complex IS*6110* on THOR after cough sampling in a prison hotspot.

Subjects	no.	Positive detection on THOR
Inmates investigated	66	12
Inmates without known history of TB	61	10/61 (16%)
Inmates with TB or prior history of TB	5	2/5 (40%)
Guards investigated	18	0/18 (0%)

## 4 Discussion

Tools that detect pathogens in air are needed to counter the spread of airborne infections but have remained absent from TB control measures, where delayed or missed diagnosis remains a problem [26]. THOR's practical, low-cost, simple design is based on the need of using it in TB hotspots. It is as such the first electrostatic air sampler developed specifically as a fieldable unit for mycobacterial detection. Our results show that this novel air sampler is capable of collecting aerosolized microparticles at low aerosol concentrations and fit for deployment in a prison hotspot, where together with PCR it identified a molecular signal for *Mtb* from patient cough.

THOR sampling is based on negative air ionization by corona discharge, with electrostatic transport and precipitation of ionized, airborne particles onto a solid collector piece, similar to large-scale electrostatic precipitators that are effectively used to filter air in industrial applications. Despite early seminal work [27,28] and more recent developments on electrostatic sampling of bioaerosols [[29], [30], [31], [32]], this technology has been poorly utilized for collecting airborne bacteria for analysis or diagnostic purposes. On the contrary, filtration-type samplers and inertial/gravitational-type samplers such as cascade impactors, impingers and cyclone samplers have seen significant use in microbiological air sampling, as well as passive gravitational-type samplers such as settle plates [33]. Both THOR and filtration/inertial-type samplers rely on an active mechanism to generate air flow for sampling. Whereas THOR uses electrostatics in an open-air environment, most filtration/inertial-type samplers use an air/vacuum pump and an integrated flow path with flow rates typically ranging from liters up to hundreds of liters per min, depending on the type of sampler and application [34].

With THOR, it is challenging to estimate the generated total flow rate of ambient air in 3D. However, the flow rate does not necessarily directly translate to bioaerosol sampling efficacy. For electrostatic samplers and unlike filtration/inertial-type samplers, electrohydrodynamic transport of ionized particles is of greater interest than the overall flow rate, which also includes neutral particles that will not be captured unless they are first ionized. Nevertheless, THOR enables easy usage, sample extraction and cleaning of contaminated surfaces as well as low power consumption, portability and silent operation, in stark contrast to the commonly used and more elaborative vacuum pump-based bioaerosol samplers. Furthermore, different types of samplers should only be compared within the scope of their intended use and application [35]. In contrast to impactors or filter-based samplers, THOR could be used to study the capture of sub-micron particles, which may be relevant for tracing genomic material in TB hotspots. Impingers and cyclone-type samplers are potentially better suited than impactors for bioaerosol sampling in conjunction with molecular methods of detection, but are relatively elaborate, require precisely-controlled air flows by vacuum pumps and are far less obtrusive compared to THOR when considered for deployment in TB hotspots.

In the context of TB, various filtration methods coupled to PCR-based detection have been used to identify airborne *Mtb* in hospitals or healthcare settings [19,20,[22], [23], [24],^36^]. More complex approaches have succeeded also in re-growing *Mtb* from patient cough or exhaled breath by using inertial/gravitational-type samplers [4,[11], [12], [13], [14], [15]]. Our results confirm the suitability of PCR to readily detect mycobacterial genomic material in air. More importantly, we extend on previous attempts to detect airborne *Mtb* by providing a novel, portable/handheld ionization and electrostatic-based solution for *Mtb* sampling that is sustainable and useful in the complex, congregate settings of TB hotspots.

The implementation of bioaerosol sampling in outbreak and pathogen surveillance investigations is growing and believed to enhance capacity-building response measures [37]. As a field-ready, scalable diagnostic, THOR would serve to screen individuals through cough or populations of individuals through environmental air sampling. This would help identify TB patients at point-of-care, screen individuals as they enter a congregate setting, identify transmission hotspots and assess the effectiveness of transmission-blocking strategies. That said, additional testing is needed to establish diagnostic ability. The THOR collector piece remains an important consideration in the above. It can easily be removed, replaced, decontaminated and reused. Material from the collector can be extracted with minimum processing into a low sample volume that keeps the sample concentrated and amenable to microbiological and molecular analyses. Furthermore, the collector piece is the point of integration to GeneXpert (Cepheid, USA) [38]. Integrating THOR with GeneXpert would reduce and automate several steps of the pathogen-detection process. It would remove the need of trained personnel and access to a laboratory to perform detection. In other words, integration with GeneXpert is a way to streamline THOR development into a standalone sampler-detector system for TB hotspots.

During THOR operation, the rapid clearance of aerosolized microspheres from the chamber was associated with an increased detection of microspheres on the THOR collector piece. Analysis of *Bg* re-growth from various parts of THOR clearly shows that the collector piece is the primary site of active sampling on the unit (Supplementary Fig. 4). Moreover, prolonging the sampling time up to 60 min improved the collection of aerosolized microspheres on THOR (Supplementary Fig. 5). Also, repeated aerosol dispersals of microspheres within a 60 min-sampling interval increased the total number of microspheres detected on the THOR collector piece (Supplementary Fig. 5), indicating that the collector piece is not saturated after 60 min of sampling with a single aerosol dispersal of microspheres. These observations suggest that the collector piece may provide sufficient surface for prolonged sampling. However, corona ionization discharge from THOR may have caused aerosolized particles to electrostatically precipitate onto other surfaces of the chamber. Although precipitation to sites other than THOR does not contribute to pathogen detection it helps nevertheless to remove infectious particles from the air. Ionization has been shown to prevent transmission of airborne TB [39] and to inactivate viruses and bacteria [[40], [41], [42]]. These observations are in line with poor re-growth of mycobacteria from THOR in our laboratory experiments. THOR may thus serve TB control measures not only by detecting *Mtb* in air, but also by purifying the air as an inherent part of its operation. This safety feature of the unit is particularly advantageous for resource-limited settings.

The low amounts of tubercle bacilli expected to occupy airspace may require collection of large volumes of air for its detection. Sensitive detection is therefore needed for monitoring *Mtb* in air. We estimated the limit-of-detection of THOR sampling on the basis of the lowest concentration of aerosolized bacilli (BCG or *Bg*) at which detection on THOR was still positive and above that of passive sampling (*i.e.* THOR *off*). The limit-of-detection for 15 min sampling for both BCG and *Bg* in our tests was around 10^5^ CFUs/mL, corresponding to approximately 11 Bg CFUs/L_air_ and 46 BCG CFUs/L_air_. Results from prison hotspots showed that 3 min sampling sufficed for detection of IS*6*110 by PCR in the cough of inmates. After treatment initiation, mycobacterial DNA in sputum has been shown to persist longer than viable bacilli [16]. This could explain the PCR-positive results from the cough of the 2 inmates who were healthy at the time of THOR sampling but that had been previously treated for TB. We speculate that THOR identified a leftover IS*6110* signature in these patients originating from bacterial presence in their previous TB episode. It is also possible that THOR can identify an *Mtb* trail from a location previously inhabited by infected occupants. In this regard, we found that THOR-sampled air from a holding cell in the infirmary ward at PED was positive for IS*6110,* while similar sampling from guard quarters off-limit to inmates, was negative.

The 10 randomly-selected inmates that tested positive on THOR did not have TB at the time of sampling nor were they diagnosed with TB during a prison-wide TB screening performed 1 year later. The limited scope of our field testing did not allow us to establish diagnostic utility of THOR cough sampling. Additional testing is needed to define detection during cough and other relevant sampling scenarios with THOR. *Mtb* in cough-generated aerosols has previously been used to identify infectious patients and to predict transmission in household contacts [13,14]. The link between positive amplification of mycobacterial DNA on THOR, active TB and bacillary status in sputum merits investigation. Unraveling this will establish how detection of an *Mtb* molecular signature in cough pans as a proxy for active TB; and in the air in hotspots as an indicator for infection risk. Both would contribute to TB control.

## Funding

This work was supported by the Bill and Melinda Gates Foundation (grant number OPP1118552), Karolinska Innovations AB, and Karolinska Institutet, all to A.G.R. The funders had no role in study design, data collection and interpretation, or the decision to submit the work for publication.

## Declaration of competing interest

The authors declare no conflicts of interest.
