# Supplementary material for: A fieldable electrostatic air sampler enabling tuberculosis detection in bioaerosols
Source: Tuberculosis (Edinb). 2020 Jan;120:101896. doi: 10.1016/j.tube.2019.101896 (PMC7049907; doi:10.1016/j.tube.2019.101896)
Supplement: Multimedia component 2 [file mmc2.docx]

**Supplementary Material**

**Supplementary Figure 1.** Visualization of electrostatic field lines and smoke sampling on THOR. **A,** Photograph of THOR showing collector piece, negative corona discharge ionizers and power socket (left panel). Cross-sectional view (A-A´) of a simplified 2D finite element model with surface (field strength) and arrow (direction) plots of the electric field generated by the potential difference between the ionizers and the collector piece (right panel). Relative field strength shown ranging from weak (blue) to strong (red). Scale bar represents 1 cm. **B,** A smoke pen was lighted, brought into proximity of the THOR collector piece (image on left). THOR was shortly thereafter activated. Rapid acceleration and redirection of the smoke stream towards the collector piece on THOR was observed (image on right). Also **Supplementary** **Movie 1**.

**Supplementary Movie 1.** Visualization of smoke sampling on THOR. Video showing sampling of smoke particulates onto the THOR collector piece in real-time. Related to **Supplementary Figure 1B**.

**Supplementary Figure 2.** Containment suite for aerosol sampling experiments. An airtight chamber made of flexible polyvinyl chloride (PVC) polymer was custom-tailored (Solo Containment) and mounted on a chromed-plated steel frame inside a Biosafety Level-2 laboratory. The enclosure has outer dimensions 270 cm (L) x 165 cm (W) x 255 cm (H) with an inner volume of 9.3 m^3^ (9300L). A BLAM aerosol generator (CH Technologies) is mounted on the right-hand side of the chamber in a central position 195 cm from the ground. A H14 HEPA filter panel is mounted on the same side on the lower bottom end (not shown). A 40 cm cubic pass box with a hand-operated 70% ethanol spray is mounted in a central position on the left-hand side of the chamber approximately 82 cm from the ground, used for removing materials from the chamber after surface disinfection. The inside of the enclosure is decontaminated with 35% hydrogen peroxide vapor with a BQ-50 unit (Bioquell), operated remotely from the outside.

**Supplementary Figure 3.** Aerosolized microspheres sampled on THOR detected by flow cytometry. **A** and **B**, FluoSpheres (1 µm, 1x10^9^) were aerosolized as in **Figure 2.** Air sampling on THOR and Coriolis done as previously described for *Bg* in **Figure 5A** and **B.** FluoSpheres sampled on Coriolis and THOR were quantified by flow cytometry. Bars indicate standard error of the mean. Data from at least 3 experimental replicates for each group are graphed. * denotes statistically significant differences between THOR and Coriolis (**A**) or between THOR *On* and THOR *Off* (**B**).

**Supplementary Figure 4.** The collector piece is the primary site for active sampling of aerosols on THOR. *Bg* spores (1.2x10^7^ CFUs) were aerosolized in the enclosure. THOR was wiped with 70% ethanol, a collector piece mounted, the unit turned *on* (THOR *On*) or left inactive (THOR *Off*). After 15 min of sampling, the collector piece was removed, sample extracted and *Bg* quantified on agar. In addition, the top surface, all four sides of the device, and the section of the electrical wire inside the aerosol chamber were swabbed, resuspended in PBS-T80 and plated on agar. Bars indicate standard error of the mean. Four experimental replicates performed and graphed. * denotes statistically significant differences between THOR *On* and THOR *Off*.

**Supplementary Figure 5.** The THOR collector piece is not saturated after 60 min of active sampling. **A**, FluoSpheres (1 µm, 1x10^8^) were aerosolized as in **Figure 2**, air sampling on THOR was performed for 5, 15, 30 and 60 min and material collected on THOR quantified at each time point by flow cytometry. **B**, FluoSpheres (1 µm, 3x10^9^) were aerosolized as in **A** and air sampling performed with THOR turned *on* (THOR *On*) or left inactive (THOR *Off*) for 60 min. Additional aerosol dispersals of FluoSpheres (1 µm, 3x10^9^) were performed every 15 min (for 4 dispersals) or 30 min (for 2 dispersals). The total amount of FluoSpheres collected on THOR at the end of the 60-min sampling interval was determined by flow cytometry. Bars indicate standard error of the mean. At least three experimental replicates for each group performed and graphed. * denotes statistically significant differences between THOR *On* and THOR *Off.* # denotes statistically significant difference between THOR-*on* mode after 1 aerosol dispersal compared to 2 or 4 dispersals.
