# Supplementary figures and images for: A fieldable electrostatic air sampler enabling tuberculosis detection in bioaerosols
Source: Tuberculosis (Edinb). 2020 Jan;120:101896. doi: 10.1016/j.tube.2019.101896 (PMC7049907; doi:10.1016/j.tube.2019.101896)

**A**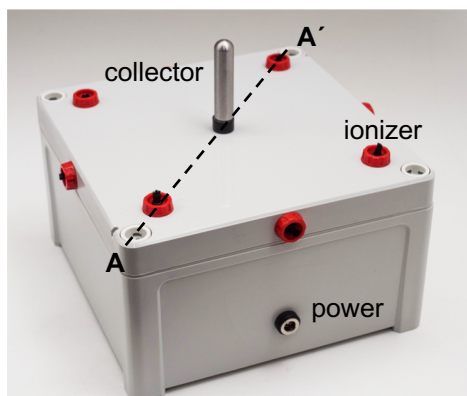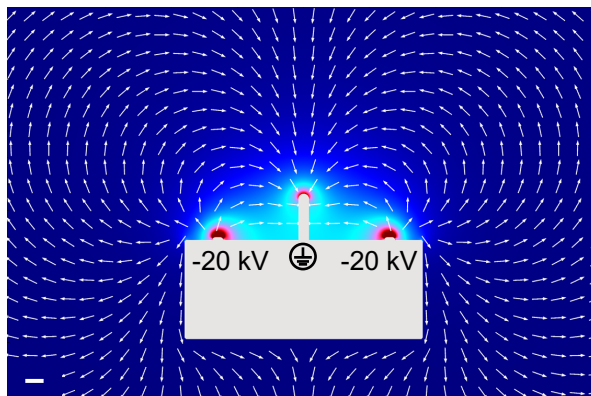**B**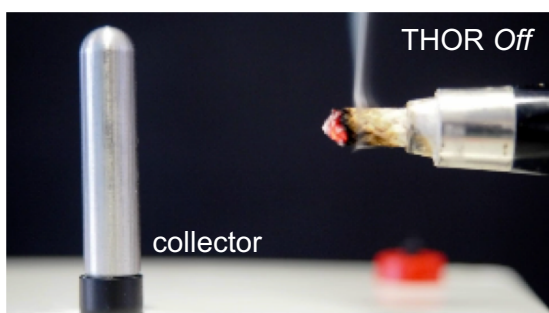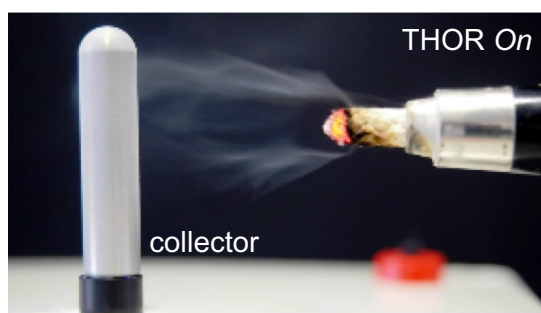

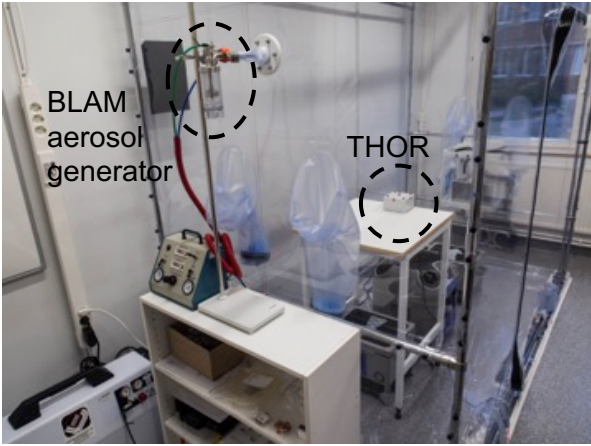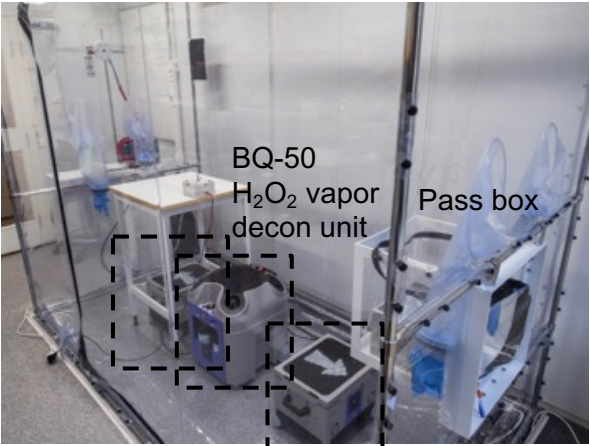

SUPPLEMENTARY FIGURE 2

**A**

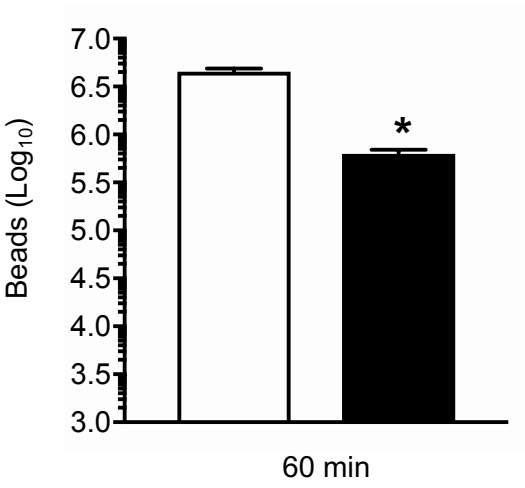

**B**

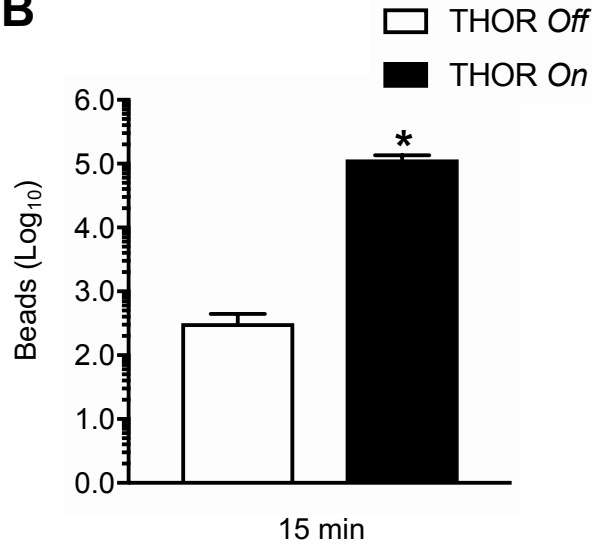

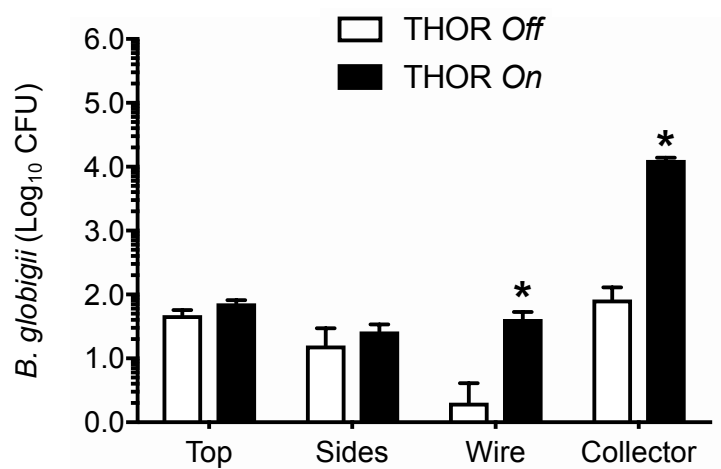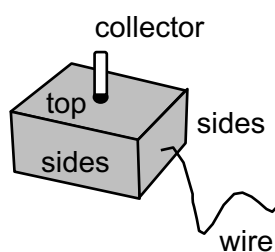

**A**

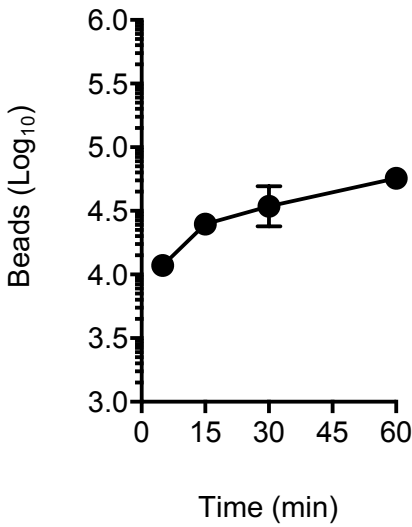

**B**

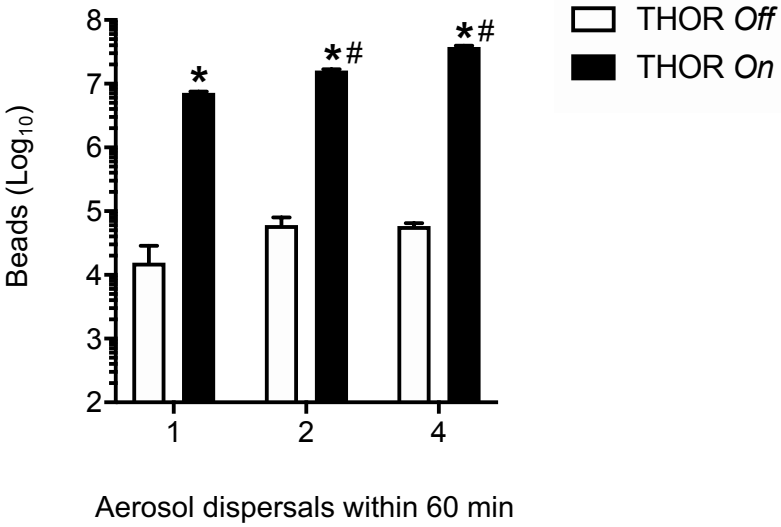

Supplement: Multimedia component 3 [file mmc3.pdf]
